# Minimal effects of oyster aquaculture on local water quality: Examples from southern Chesapeake Bay

**DOI:** 10.1371/journal.pone.0224768

**Published:** 2019-11-07

**Authors:** Jessica S. Turner, M. Lisa Kellogg, Grace M. Massey, Carl T. Friedrichs

**Affiliations:** Virginia Institute of Marine Science, William & Mary, Virginia, United States of America; Bigelow Laboratory for Ocean Sciences, UNITED STATES

## Abstract

As the oyster aquaculture industry grows and becomes incorporated into management practices, it is important to understand its effects on local environments. This study investigated how water quality and hydrodynamics varied among farms as well as inside versus outside the extent of caged grow-out areas located in southern Chesapeake Bay. Current speed and water quality variables (chlorophyll-a fluorescence, turbidity, and dissolved oxygen) were measured along multiple transects within and adjacent to four oyster farms during two seasons. At the scale of individual aquaculture sites, we were able to detect statistically significant differences in current speed and water quality variables between the areas inside and outside the farms. However, the magnitudes of the water quality differences were minor. Differences between sites and between seasons for water quality variables were typically an order of magnitude greater than those observed within each site (i.e. inside and outside the farm footprint). The relatively small effect of the presence of oysters on water quality is likely attributable to a combination of high background variability, relatively high flushing rates, relatively low oyster density, and small farm footprints. Minimal impacts overall suggest that low-density oyster farms located in adequately-flushed areas are unlikely to negatively impact local water quality.

## Introduction

Shellfish aquaculture is an important and rapidly growing industry with opportunity for continued expansion worldwide [1]. In global food production, cultured bivalves have a low environmental impact per gram of protein produced, compared with finfish aquaculture, most capture fisheries, and terrestrial livestock [2]. In the U.S., oysters are the largest grossing marine species group for U.S. aquaculture, valued at $192 million in 2016 [3]. On the Atlantic coast of the U.S., shellfish aquaculture growth and expansion can be controversial. Growers focus on the potential environmental benefits of aquaculture and its contribution to sustainable food production, while other stakeholders voice concern over viewshed, navigation, and possible negative water quality and sediment impacts.

Chesapeake Bay serves as a relevant regional example of shellfish aquaculture development amid controversy, as oyster aquaculture is an important part of the Chesapeake Bay economy and is becoming integrated into watershed management practices. In 2017 alone, intensive Virginia oyster aquaculture contributed approximately $14.5 million to the state’s economy [4], including ~130 working jobs in rural areas. In an era of declining wild populations [5,6] and expanding but costly reef restoration [7–9], farmed oyster production is becoming increasingly important. Additionally, oyster aquaculture has been partially approved as an alternative management practice for nutrient reduction in the Chesapeake Bay region [10]. As this industry grows and integrates into management, stakeholders need a greater understanding of farms’ benefits and impacts on local ecosystems.

Water quality can be improved by oyster filtration. Oysters filter sediments, detritus, small phytoplankton, and particulate-bound nitrogen and phosphorus from estuarine waters [11–13]. On average, one *Crassostrea virginica* individual market-sized oyster (~1 gram dry weight) can filter approximately 6.8 liters/hour, up to 163 liters/day in the summer at 20°C [14]. The eastern oyster has the ability to ingest tiny particles (~2–38 μm) and selectively choose food particles [13–20]. When oyster filtration is added to small-scale ecosystem models and large-scale hydrodynamic models, results include clearer water, deeper light penetration, and greater light availability to submerged aquatic vegetation [21,22].

Water quality can also be degraded by oysters. Oysters directly release ammonia into the water column via excretion, sometimes in substantial quantities [23,24]. Excretion of ammonia by oysters is of concern because it can boost the local growth and regeneration of phytoplankton [25], potentially enhancing eutrophication in summer. However, the flux of ammonia from oyster excretion to the water column has been found to be minor compared with the flux of nutrients released from oyster-associated sediments, especially sediments experiencing organic matter loading from oyster fecal production [24,26–28]. Farming oysters in high densities also introduces the potential for organic enrichment of the benthos [29]. Through production of two kinds of biodeposits, feces and pseudofeces, oysters can increase deposition of organic particles to the seafloor [25]. High volumes of biodeposits were measured at some Japanese and European oyster farms with high culture densities, causing sediment organic enrichment and oxygen depletion [30–35], likely due to farms’ poorly flushed locations [36].

Oyster aquaculture has the potential to alter hydrodynamic flow due to the position, size, and density of shellfish aquaculture gear. For example, current speed was found to be slower within many types of aquaculture operations, including scallop-kelp farms in China [37], floating scallop farms in Nova Scotia, Canada [38], mussel farms in New Zealand [39,40], and mussel raft culture in South Africa [41,42]. In marine research using other structures of comparable size to the oyster cages in this study, such as clam pens and predator exclusion cages, currents were slowed to the point where sediment deposition was increased [43,44]. Hydrodynamic effects in general depend on the porosity of the cage or gear, the spacing of the gear, and the location of the gear in the water column [45].

This study fills gaps in knowledge of aquaculture impacts by quantifying water quality effects *in situ* at multiple operating commercial farms with different spatial scales and gear types. To date, the few *in situ* field efforts to measure water quality at operational oyster farms have taken place in regions with exposed coastlines and relatively sparse human populations, for example, eastern Nova Scotia, Canada, and rural southwestern Australia [38,46]. In contrast, the Chesapeake Bay watershed is home to over 18 million people [47] and has a history of human land use change resulting in estuarine eutrophication [48]. Compared with aquaculture in other regions, oyster aquaculture in the Chesapeake Bay is not only an economic concern, but a watershed management concern and work is needed to quantify the impacts of oyster aquaculture in this region. Many past studies of oyster aquaculture have focused on laboratory, mesocosm, and modeling studies [25,49,22,37,50–53]. The present study builds upon past work by focusing on the *in situ* effects of the oyster farms and by sampling at four operating commercial farms differing in the type of gear used (floating and bottom cages), their spatial scales, and the number of oysters produced annually.

The main objective of this study was to examine four operating commercial aquaculture sites and quantify the positive or negative impacts of farms on the local water quality. A secondary objective was to broadly quantify the amount of potential filtration by oysters, in terms of total volume of water, in order to provide additional context for our results. This study hypothesized that water quality within the area containing cages (hereafter “inside”) would be significantly different from water quality outside of the extent of the cages (hereafter “outside”).

## Methods

### Study sites

**Fig 1:**
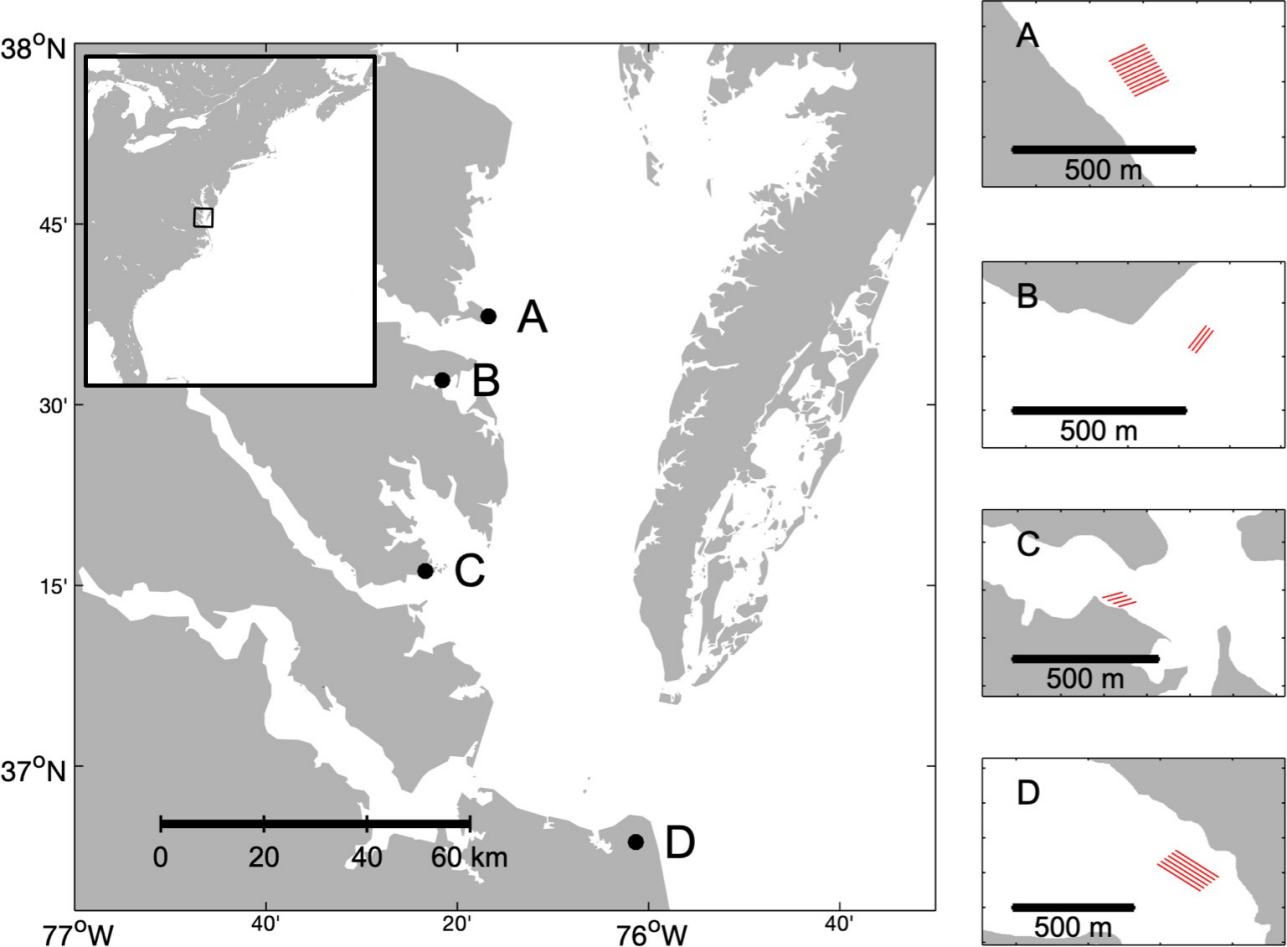
Map of study sites. The aquaculture sites sampled 2017–2018, from north to south: Windmill Point (A) near the mouth of the Rappahannock River; Bland Point (B) in the Piankatank River; Monday Creek (C) in the marshes bordering the southwestern entrance to Mobjack Bay and the mouth of the York River, and Broad Bay (D) in the Lynnhaven River system. Red lines indicate spatial extent and orientation of the cages at each site but do not correspond directly to rows in the array of cages at each site.

Data were collected at four commercial oyster aquaculture sites in the southwestern portion of Chesapeake Bay (Fig 1). From north to south, the sites included Windmill Point (37.622 N, -76.279 W), Bland Point (37.534 N, -76.359 W), Monday Creek (37.263 N, -76.389 W), and Broad Bay (36.895 N, -76.023 W). Two of the farms used floating cages, and the other two used bottom cages (Table 1). All four sites were visited in summer 2017, and the two largest sites Windmill Point and Broad Bay were sampled again in fall 2017. Permission to access the aquaculture sites was given directly by growers.

**Table 1:** Study sites.

Code (Fig 1)	Site	Gear type	Seasons sampled	Extent of cages (m^2^)	Bed composition
A	Windmill Point	Floating	Summer/Fall 2017	16200	Gravelly sand
B	Bland Point	Bottom	Summer 2017	1100	Sand
C	Monday Creek	Floating	Summer 2017	5500	Muddy sand
D	Broad Bay	Bottom	Summer/Fall 2017	39600	Sand

Characteristics of the commercial aquaculture sites visited in this study, including gear type, seasons visited, extent of cages in terms of two-dimensional area, and bottom sediment type.

Aquaculture sites differed in environmental setting but were similar in depth and salinity. The two northern sites were located in areas with greater fetch near deeper, wider channels than the two southern sites. Windmill Point was situated at the end of a peninsula exposed to the mainstem of the Chesapeake Bay. Bland Point lay in a broad open area near the mouth of the Piankatank River. Both Monday Creek and Broad Bay were located in more protected inlets. All sites had mesohaline salinities (ranging from 15–22 psu) and mean water depths of ~1 m (ranging from 0.5 to 2 m depending on distance from shore and tidal stage).

### Sediment characterization

Prior to the start of water quality sampling, sediments were collected and characterized at each site to serve as an integrated measure of local hydrodynamic regimes and to provide a broader context for results of subsequent water quality sampling cruises. Sediments were sampled in spring 2017 at Windmill Point, Bland Point, Monday Creek, and Broad Bay (n = 25 to 50 point samples per cruise). A PONAR grab sampler was used to collect sediment samples from the top ~ 2–5 cm of the bed. Sediment grain size was determined using wet sieve and gravimetric pipette analysis. The finer two size classes (< 63 μm) were defined using a nominal size (8-phi or 4-phi) and the coarser two size classes were defined in terms of a range of sizes (63–850 μm sand; >850 μm gravel and debris). Percent sand and larger and percent fine material was then quantified as the percent of all sediment by dry weight that was greater than and less than 63 μm in size, respectively. Percent organic was quantified as the percent of all sediment by dry weight that was volatized at 550°C. Sediment characteristics inside and outside farm footprints were compared using one-way analyses of variance (ANOVA) at each site, adjusting for multiple comparisons using Tukey’s honestly significant difference procedure.

### Water quality sampling cruises

**Fig 2:**
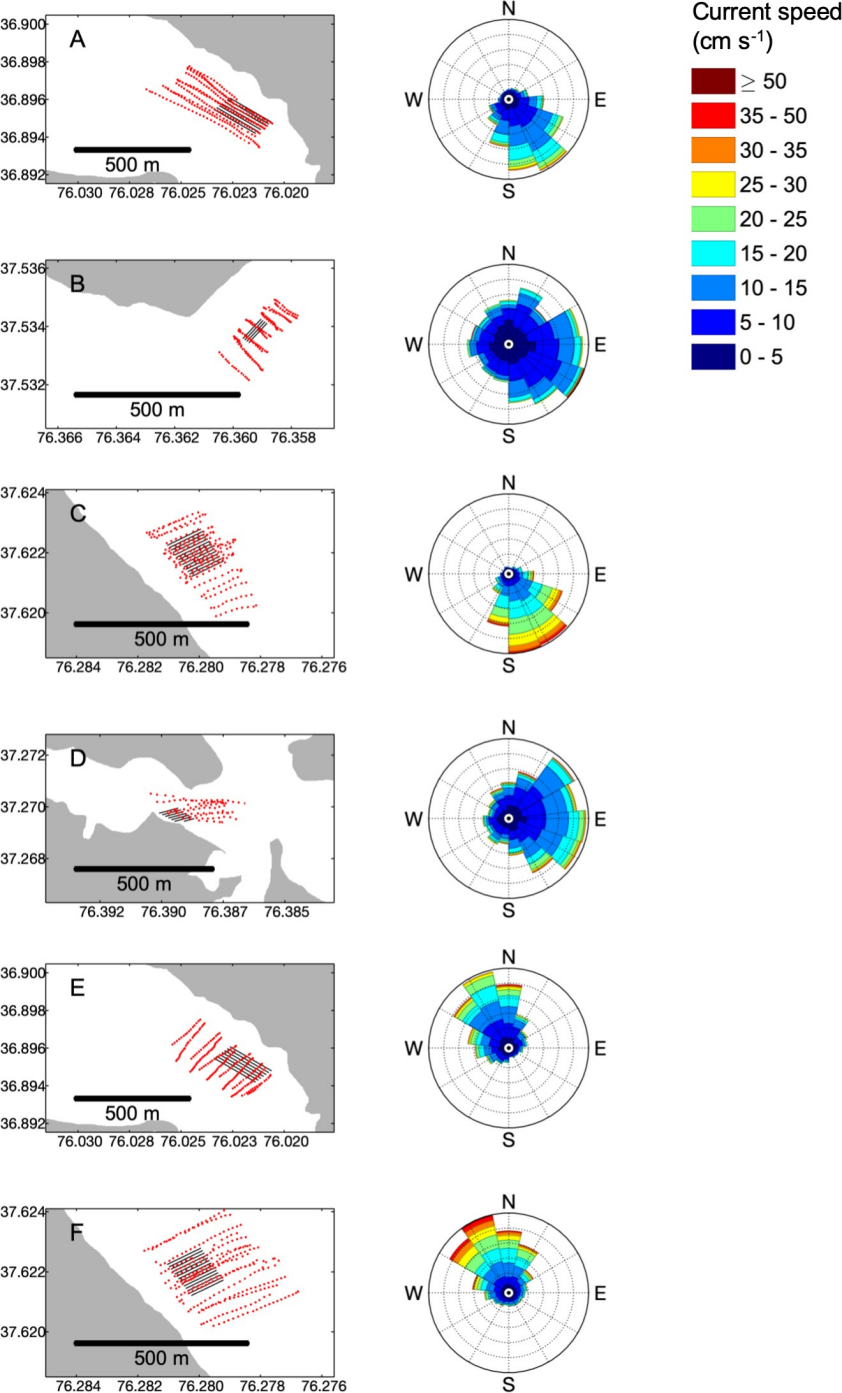
Spatial resolution of sampling and current direction on each sampling cruise. For sampling cruises at (A) Broad Bay in summer, (B) Bland Point in summer, (C) Windmill Point in summer, (D) Monday Creek in summer, (E) Broad Bay in fall, and (F) Windmill Point in fall, red circles indicate locations sampled using moving-vessel transects with every nth point shown to represent the subsampling interval for chlorophyll on each cruise, where n = 4 to 14 depending on the cruise (Table 2). Black lines indicate spatial extent and orientation of the cages at each site but do not correspond directly to rows in the array of cages at each site. Righthand panels depict polar histograms of current directions measured during each sampling cruise, with distance from the center indicating relative frequency and color indicating the proportion of each directional observation that fell within the given speeds (cm s^-1^).

Water quality variables were measured inside and outside farm areas at all sites. High frequency water quality, current speed, and location data were collected from a moving vessel along multiple transects through, upstream, and downstream of each site (Fig 2), with the total number of transects scaled to the area of the farm footprint. Cruises were designed to compare water quality outside of the extent of the cages to inside, where waters were most likely to be impacted by oyster filter feeding, excretion, and biodeposition. In addition to assessing differences between the areas inside and outside of each farm, this approach also allowed for the assessment of the scale of these differences in relation to differences between sites and seasons. Data were collected on six cruises, resulting in six separate sampling periods used for statistical analysis.

Water quality sampling cruises measured current speed and water clarity variables while the vessel was underway. The vessel was driven slowly along 10–30 transects, with the number of transects depending on the size of the farm. Roughly half of the transects crossed through the farm, while the other half were driven entirely outside (Fig 2). Because all transects started outside the farm area, all transects included at least some “outside” data (Fig 2). Sampling took place within the two to four hours bracketing predicted maximum tidal current, which included periods of time with both relatively slow and relatively fast current speeds. During each transect, an RDI acoustic Doppler current profiler measured current speed and direction. A YSI 6600-series sonde measured temperature, salinity, chlorophyll-a fluorescence (henceforth “chlorophyll”), turbidity, and dissolved oxygen (DO). In addition to moving vessel transect flow measurements at Windmill Point in summer, long-term flow measurements collected by a stationary upward-facing acoustic doppler profiler (ADP) deployed just outside the farm footprint over 31 days. The ADP was deployed August 8 to September 8, 2017 and collected data every 15 minutes. Within that timeframe, Windmill Point summer transects were sampled on August 31, 2017, collected data approximately every second for four hours.

### Statistics

**Fig 3:**
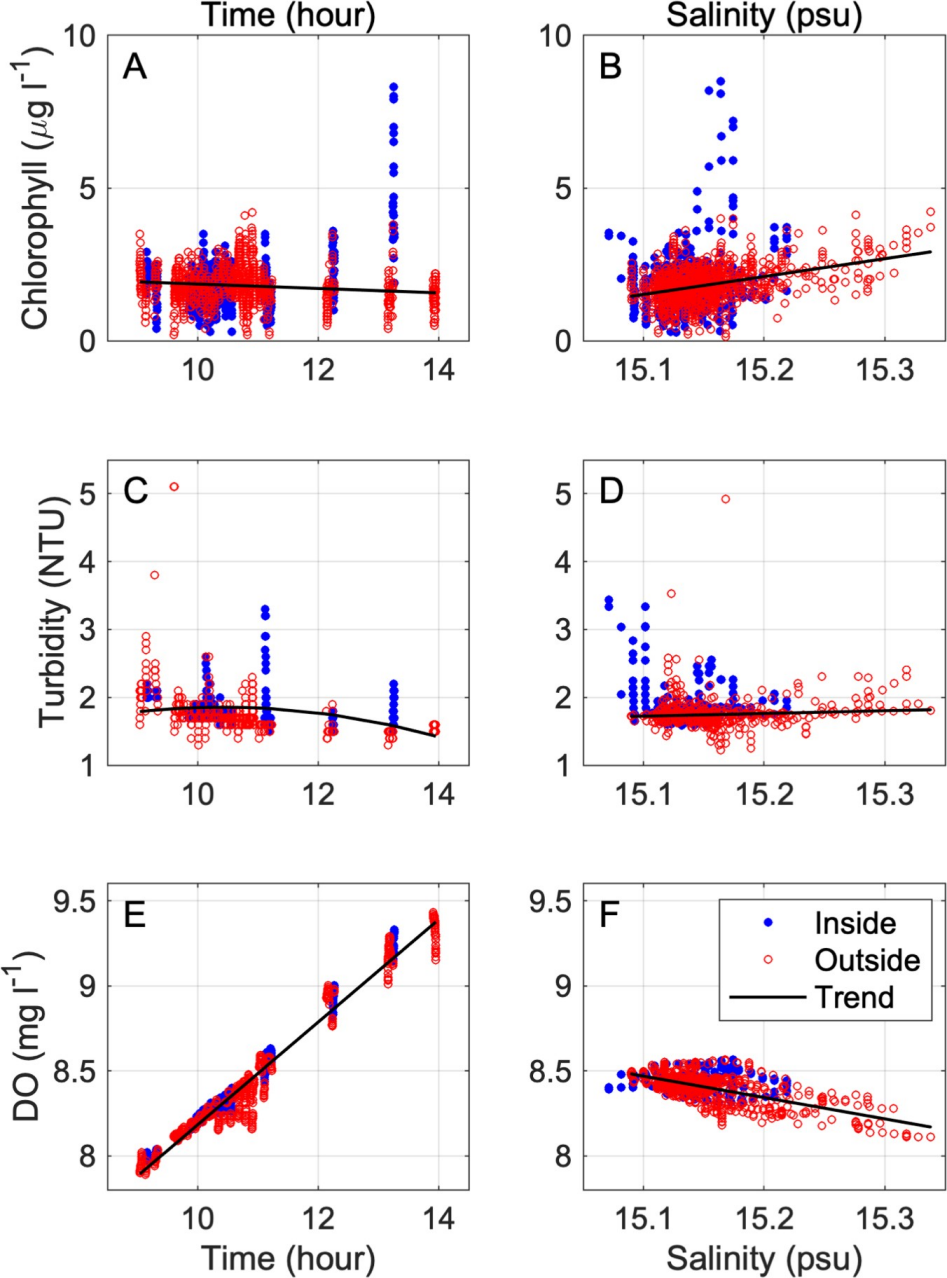
Water quality detrending process for transect data. To account for trends associated with time of day, such as warming temperatures throughout the day, water quality data were detrended with respect to (A, B, C) time and (D, E, F) salinity. Red circles indicate points sampled inside and blue circles indicate points sampled outside the farm footprints. Current speed was detrended with respect to time, but not salinity.

All of the variables we measured were expected to change across the entire site due to tides and changes in solar irradiance throughout the day. To increase our ability to detect changes between waters inside and outside the farm footprint (hereafter “farm effect”), all data that showed significant patterns with respect to time and salinity were detrended. Before detrending, data were classified as “inside” or “outside” data based on whether they were collected inside the footprint of the farm or outside of that footprint. Outside data included data from transects run entirely outside the farm and data from the portions of transects running through the farm that fell outside the farm footprint. Data that fell more than four standard deviations away from the mean of outside data were removed as outliers prior to detrending. For all variables of interest, detrending (Fig 3) was accomplished by plotting data collected outside the farm footprint against time and identifying the polynomial best fit regression. For current speed data, if a second-order polynomial fit as a function of time was significant (α = 0.05), the outside-farm trend was subtracted from the full dataset (i.e. data from inside and outside the farm footprint). For water quality data, the best fit of three regressions–linear, 2^nd^-order polynomial, and 3^rd^-order polynomial–was used to detrend each variable over time for each cruise. The best fit was determined based on the relative change in the R value between different regressions. In most cases a linear fit was the most effective method for detrending variables over time (Fig 3). To better visualize farm effects after detrending relative to the original magnitudes of the variables at each site, residuals after detrending were scaled back to the original outside-farm means. This was done by adding a constant to the full (inside and outside farm) detrended data set for each variable at each site such that the mean values for variables sampled outside the farm footprints were made equal to their original mean values.

**Fig 4:**
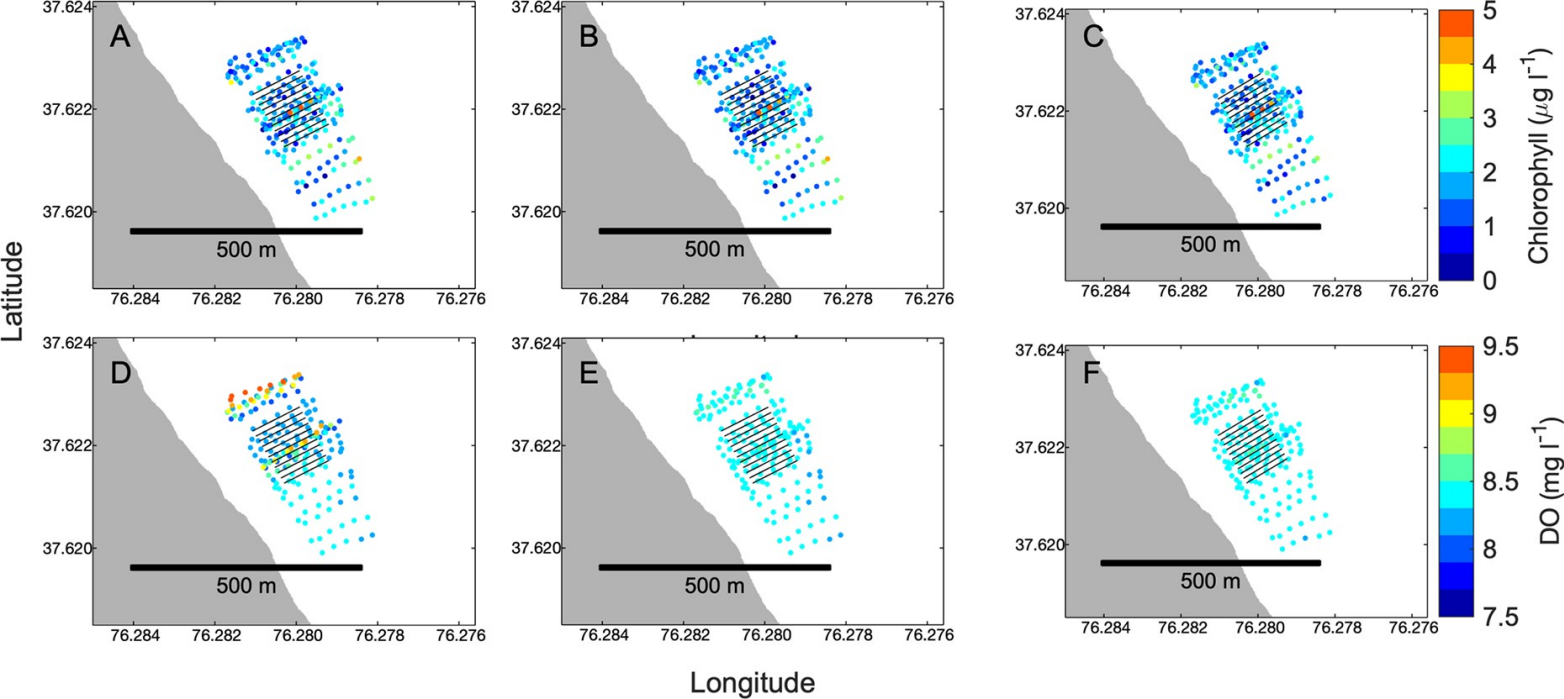
Examples of water quality measurements before and after detrending. On the top row, the color of points indicates chlorophyll concentration (μg l^-1^) at Windmill Point in summer 2017, (A) before detrending for either time or salinity, (B) after detrending for time, before detrending for salinity, and (C) after detrending for both time and salinity. On the bottom row, the color of points indicates DO concentration (mg l^-1^) (D) before detrending, (E) after detrending for time, and (F) after detrending for both time and salinity. For chlorophyll every 7^th^ point is shown, and for DO every 9^th^ point is shown, corresponding with the subsampling interval for those variables on this cruise (Table 2).

In most cases, the spatial pattern of water quality was not strongly influenced by the detrending procedure. For example, change in the spatial distribution of chlorophyll at Windmill Point in summer before and after detrending was negligible (Figs 3A, 4A, 4B and 4C). However, for some variables on certain cruises, the original spatial pattern of variability was strongly confounded by temporal trends such as warming throughout the day of sampling, such as the observed pattern of DO at Windmill Point in summer (Figs 3E, 4D, 4E and 4F). For cases such as summer DO, removing the trends associated with time and salinity allowed for a rigorous comparison of water quality inside vs. outside cages. Further examples of data before and after removal of outliers and detrending are graphically displayed in this study’s data repository [54].

Once data were detrended, they were subsampled according to the number of sampling points over which observations were autocorrelated in space for each variable on each cruise. Using measurements outside of cages, each transect was fitted to a linear trend, which was subtracted from the transect. An autocorrelation analysis was then performed on the outside data along each transect in order to calculate the “limiting lag” at which the data were no longer spatially autocorrelated with one another [55]. The median limiting lag characterizing each variable on each cruise was then determined (Table 2). Last, the measurements of each variable from each cruise were subsampled according to their respective limiting lags, using random start points. Full details of the autocorrelation analysis used to determine subsampling intervals can be found in this study’s data repository [54].

**Table 2:** Median limiting lags used for subsampling for each variable on each cruise.

Site	Season	Median Limiting Lag			
		Current Speed	Chlorophyll	Turbidity	DO
Windmill Point	Summer	1	7	4	9
	Fall	2	8	7	14
Bland Point	Summer	2	4	7	5
Monday Creek	Summer	3	14	11	12
Broad Bay	Summer	2	9	15	14
	Fall	2	6	10	12

The median limiting lag for a given variable on a given cruise was determined by the autocorrelation function, and represents the characteristic number of points over which that measurement was autocorrelated in space.

Once data were subsampled, a combination of 2- and 3-way analyses of variance (ANOVA) were used to assess the effects of location with respect to the farm footprint (inside vs. outside), site, season, and their interactions on current speed, chlorophyll, turbidity, and DO. A two-way factorial ANOVA with four levels of site (i.e. Windmill Point, Bland Point, Monday Creek, and Broad Bay), and two levels of location relative to farm (inside vs. outside) was used to assess the effects of these factors on data collected in summer 2017. We then assessed the effects of season in addition to other factors using a three-way factorial ANOVA with two levels of site (Windmill Point and Broad Bay), two levels of season (summer and fall), and two levels of location relative to farm (inside vs. outside). When significant interactions between terms were identified, additional analyses were carried out within levels of factors and factor combinations as appropriate. When a factor was identified as significant, post-hoc testing (Holm-Sidak) was used to identify significant differences between levels of that factor. Significance for all statistical tests was set to α = 0.05. In cases where data failed to meet ANOVA assumptions of normality and/or equal variance and were resistant to transformation, ANOVA were assumed to be robust to these violations. ANOVA results are presented in full in this study’s data repository [54].

### Conceptual farm-scale filtration calculation methods

To complement observational measurements of water quality at aquaculture sites, a simplified conceptual model was used to evaluate the potential for filtration by oysters at each farm. The following equation was used to estimate the fraction of the total water volume passing through each farm that could be filtered by oysters on a given flood or ebb tide:

$$Fraction\;of\;water\;filtered\approx \frac{\left(TNF\right)}{V}$$

where *T* is the time passing water is exposed to oysters based on flow distance divided by the current speed, *N* is the number of oysters, *F* is the mean maximum filtration rate of the oysters (about 1x10^-6^ m^3^ s^-1^ oyster^-1^) based on a review of studies [56], and *V* is the volume of the 3D-box-shaped aquaculture site within the extent of the cages, based on its dimensions measured using GIS. Current speeds were based on speeds calculated from north- and east-velocity components observed at each of the four sites sampled in summer 2017, and numbers of oysters were estimated based on growers’ reported annual harvests and approximate observed numbers and sizes of cages present at each site during summer 2017 [57,58]. This simplified calculation makes a variety of assumptions, including consistent current velocity through the site without consideration of lateral mixing, adult oysters, constant filtration rate, and an even distribution of water contact with oysters. This calculation likely overestimates filtration rates, because the filtration rate applies to summer temperatures for adult oysters, and the calculation does not account for refiltration, such as the refiltration that may occur if oysters filter the same parcel of water during consecutive flood and ebb tides. Though simplified, this calculation provides a context for the results observed *in situ*.

## Results

Our studies of oyster aquaculture farms in Virginia found that, although farms had statistically significant effects on the environmental variables measured, those effects generally were small in scale. Differences among farms and among seasons were generally of a far greater magnitude than differences between areas inside and outside of the farm footprint within individual sites. In addition, the magnitude and direction of the effect of farms on environmental variables varied in complex ways with site and season. Significant interactions between factors were common, requiring analyses of each factor within combinations of the other factors of interest.

### Sediment characteristics

Sediment grain size and organic content were homogenous at each site, with no significant differences between inside and outside of cages (p > 0.05) at most sites with the exception of Windmill Point, where sediment organic content was slightly higher outside of cages (difference of 0.3%, p = 0.034). Bed composition was used as a proxy for the wave exposure at each site, which was not directly measured (Table 3).

**Table 3:** Site characterization of sediment bed composition.

Site	Sample Size	% Sand and larger	% Fine	% Organic	Wave exposure
Windmill Point	44	97.8	2.2	0.7	High
Bland Point	41	95.1	4.9	0.9	Moderate
Monday Creek	26	57.1	42.9	6.1	Very low
Broad Bay	25	94.0	6.0	1.3	Low

Percent sand and larger vs. percent fine material is the percent of all sediment by dry weight that was greater than vs. less than 63 μm in size, respectively. Percent organic was the percent of all sediment by dry weight that was volatized at 550°C.

### Farm and site effects

**Fig 5:**
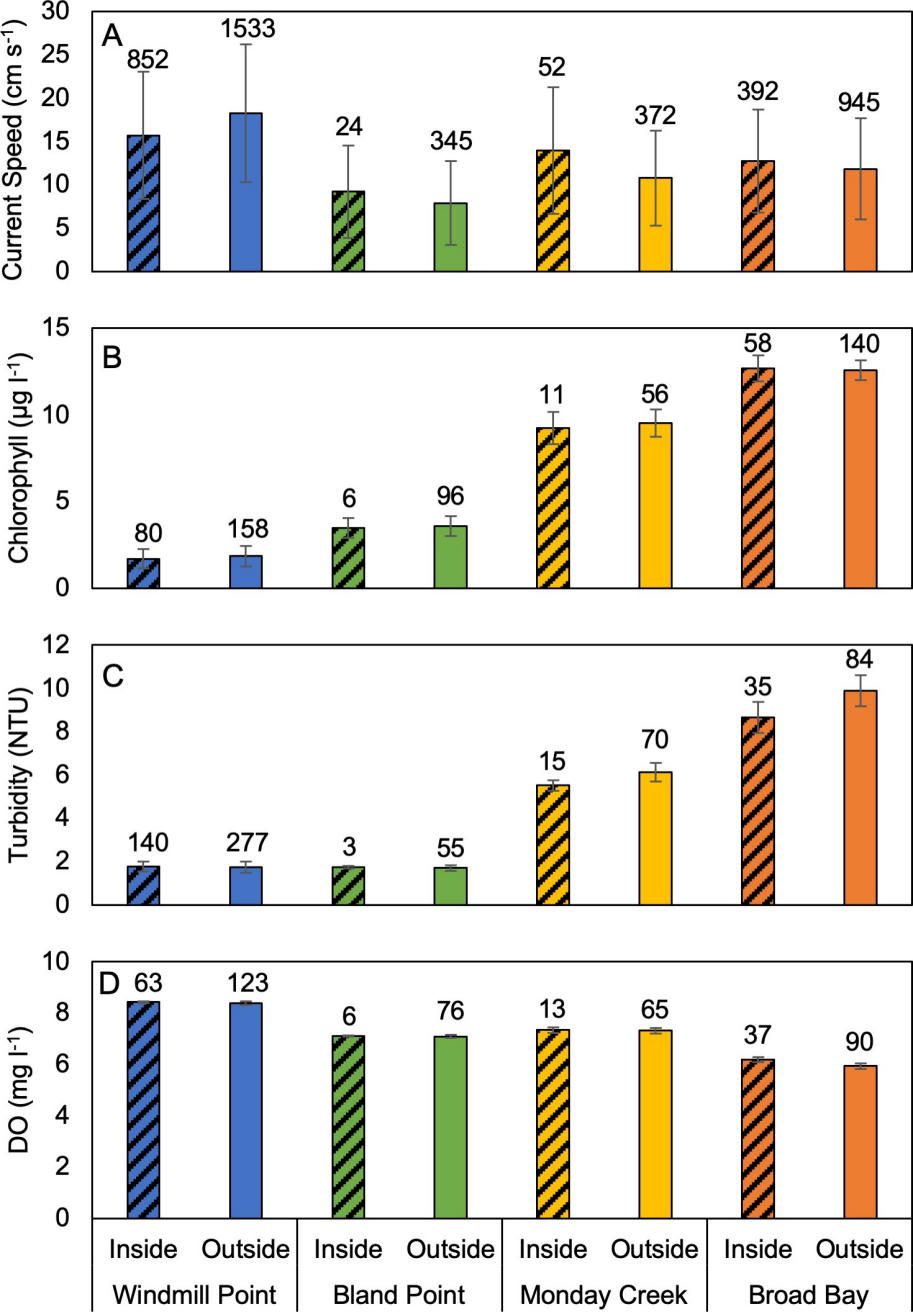
Site comparison of current speed and water quality and during summer 2017. (A) Current speed, (B) chlorophyll, (C) turbidity, and (D) DO. Error bars indicate ± one standard deviation. Sample sizes are shown above each bar. Note that for sites with large sample sizes, α = 0.05 confidence bounds on the means (not shown) are much smaller than the standard deviations.

Data were collected at all four aquaculture sites during summer 2017, enabling analysis of the effects of location relative to farms (i.e. inside or outside the farm footprint) and site on current speeds and water quality variables (Fig 5). Across all variables measured, the greatest differences were attributable to site effects rather than farm effects. For current speeds, there was a significant interaction between the effects of site and farm (p < 0.001) requiring assessment farm effects within each site and assessment of the effect of site within each level of location relative to farms (i.e. inside and outside the farm footprint). Current speeds were significantly higher inside the farm at Monday Creek and Broad Bay (p = 0.003 and p = 0.024, respectively), significantly lower inside the farm at Windmill Point (p < 0.001), and were not significantly different between inside and outside the farm at Bland Point (p = 0.25). For areas outside the farms, all sites had significantly different current speeds (Windmill Point > Broad Bay > Monday Creek > Bland Point). For areas inside the farm footprint, most sites were significantly different from one another (Windmill Point > Broad Bay = Monday Creek > Bland Point). The magnitude of the effect of the farm on current speeds ranged from 0.9 to 3.0 cm s^-1^ whereas the effect of site on current speed ranged from 1.1 to 10.5 cm s^-1^ (outside data).

For chlorophyll, there was not a significant interaction between farm and site effects (p = 0.22). Location relative to farm had no effect on chlorophyll (p = 0.13) but the effect of site was highly significant (p < 0.001). All sites were significantly different from one another (Broad Bay > Monday Point > Bland Point > Windmill Point), with the magnitude of differences between site means ranging from 1.8 to 10.8 μg l^-1^ for data collected outside the farm.

For turbidity, there was a significant interaction between farm and site effects. Turbidity was significantly lower inside the farm than outside the farm at Broad Bay and Monday Creek (p < 0.001 respectively). Regardless of location relative to farm, there were significant differences in turbidity between all sites except Windmill Point and Bland Point (Broad Bay > Monday Creek > Windmill Point ~ Bland Point). The magnitude of differences in turbidity between sites (3.7–8.2 NTU) was much greater than the magnitude of differences between samples collected inside and outside the farm (0.7–1.2 NTU).

As for turbidity, there was a significant interaction between farm and site effects on dissolved oxygen (p < 0.001). At Broad Bay, DO was significantly higher inside the farm than outside the farm (p < 0.001). At all other sites, location relative to farm did not have a significant effect on dissolved oxygen levels. Regardless of location relative to farm, there were significant differences in DO between all sites (Windmill Point > Monday Creek > Bland Point > Broad Bay). The magnitude of difference in DO between inside and outside the farm at Broad Bay (0.2 mg l^-1^) was equal to or as much as an order of magnitude less than the magnitude of differences between sites (0.2–2.5 mg l^-1^).

### Seasonal effects

**Fig 6:**
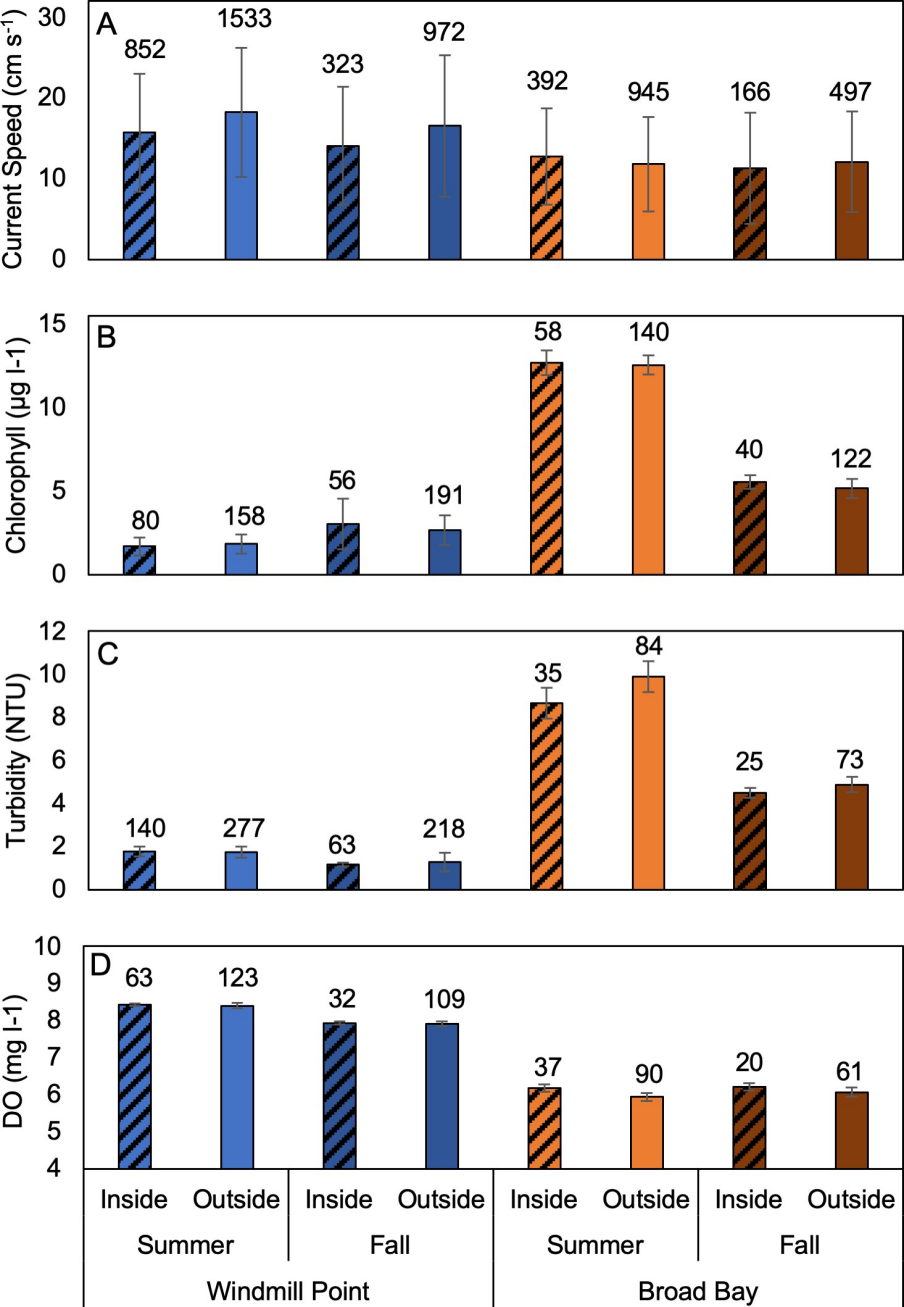
Seasonal comparison of current speed and water quality at two sites. (A) Current speed, (B) chlorophyll, (C) turbidity, and (D) DO. Error bars indicate ± one standard deviation. Sample sizes are shown above each bar.

At two sites, Windmill Point and Broad Bay, sampling was conducted during both summer and fall 2017, allowing us to examine the effects of season in addition to the effects of farm and site (Fig 6). For current speed, there was a significant interaction between the effects of site and season (p = 0.014) but no interaction between farm effects and other factors. At Windmill Point, current speeds were significantly lower in fall than in summer (p < 0.001). At Broad Bay, current speeds were similar between seasons (p = 0.10). As seen previously for summer, there were significant differences between sites in the fall (p < 0.001). In contrast to previous analyses, a significant effect of the farm on current speeds was only found at Windmill Point (p < 0.001). The magnitude of differences in current speed between seasons at Windmill Point (1.8–1.9 cm s^-1^) was slightly less than the magnitude of differences between areas inside and outside the farm (2.4–2.5 cm s^-1^), which was roughly half the magnitude of the differences in current speeds between the two sites (2.8–6.6 cm s^-1^).

For chlorophyll, there was a significant interaction between the effects of farm and season (p < 0.001) but no interaction between site effects and other factors. Broad Bay had significantly higher chlorophyll than Windmill Point, regardless of season or location relative to the farm (p < 0.001). Season had significant but opposite effects on chlorophyll concentrations at the two sites with higher chlorophyll at Broad Bay in summer (p < 0.001) and higher chlorophyll at Windmill Point in fall (p < 0.001). In contrast to summer, chlorophyll concentrations were significantly higher inside the farm at Broad Bay in fall (p < 0.001) but inside-outside differences (0.05–0.4 μg l^-1^) were an order of magnitude lower than the differences attributable to the effects of site (2.6–10.8 μg l^-1^) and season (0.8–7.4 μg l^-1^).

For turbidity, there were significant interactions between all factors (p < 0.001). At Windmill Point, the farm had no effect on turbidity regardless of season. At Broad Bay, turbidity inside the farm was significantly lower in both fall and summer (p < 0.001). At both sites, turbidity was lower in fall than in summer (p < 0.001). In both seasons, turbidity was lower at Windmill Point than at Broad Bay (p < 0.001). The magnitude of the effect of season on turbidity (0.45–5.0 NTU) was comparable to the range of magnitude of inside-outside farm effects (0.07–0.38 NTU) and site effects (3.3–8.2 NTU).

DO also had significant interactions between all factors (p < 0.02). At Broad Bay, DO was higher inside the farm than out in both summer and fall (p < 0.001). At Windmill Point, the farm had no effect on DO regardless of season. At Broad Bay outside the farm, DO was higher in fall than in summer (P < 0.001) but season had no effect on DO inside the farm. At Windmill Point, DO was higher in summer than in fall (p < 0.001) while at Broad Bay it was lower in summer, but only outside the farm (p < 0.001). Regardless of season and location relative to the farm, DO levels were higher at Windmill Point than at Broad Bay (p < 0.001). Overall, the effect of site (1.7–2.5 mg l^-1^) on DO was an order of magnitude greater than the seasonal effects (0.03–0.5 mg l^-1^) and inside-outside farm effects (0.02–0.2 mg l^-1^).

### Farm effects with distance

**Fig 7:**
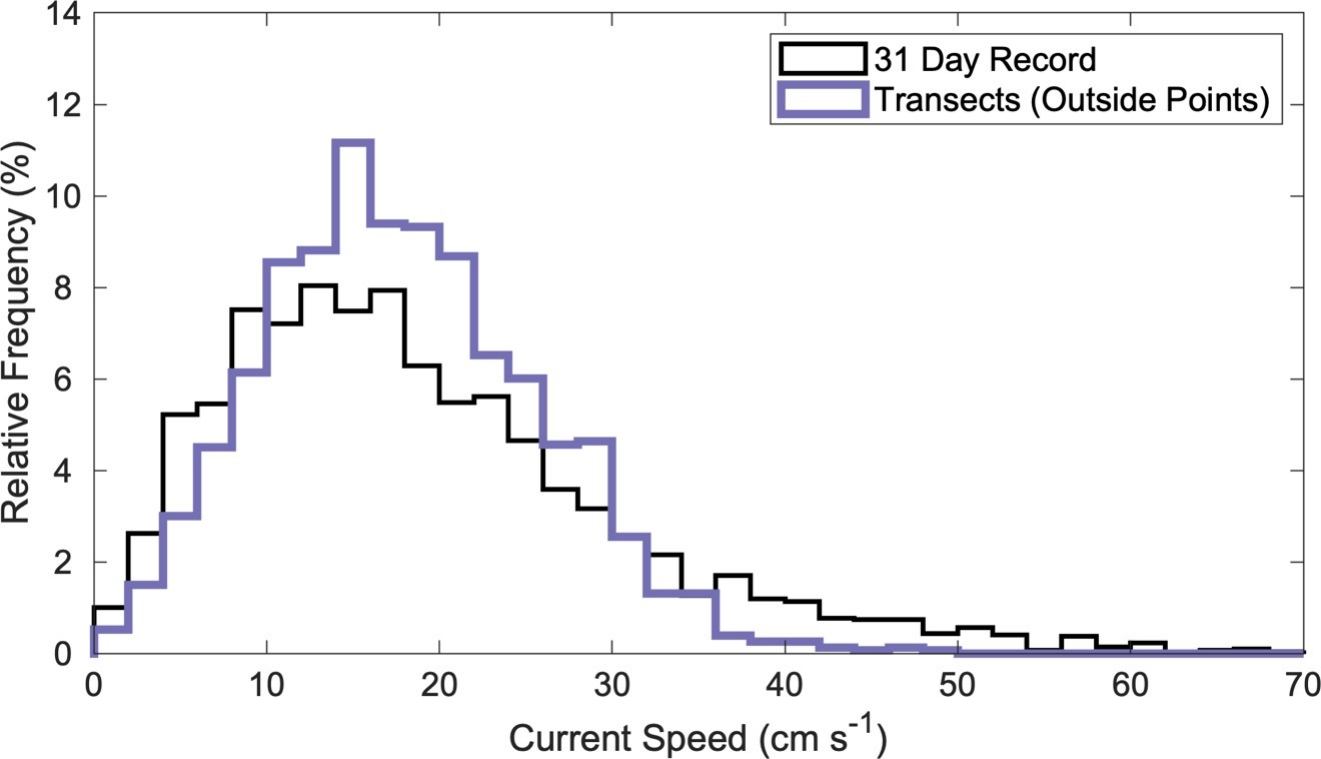
Current speeds on the Windmill Point summer sampling cruise compared to the one-month record. Transect measurements from outside the farm footprint (thick purple line) compared to the measurements collected by the ADP (thin black line).

Only one site, Windmill Point, was suitable for detailed analysis of how water quality and current speed changed as a function of distance from the upstream end of sampling. Due to complex bathymetry and the proximity of adjacent shorelines, the other sites were not appropriate for this type of analysis. At Windmill Point, bathymetry was more uniform, and current direction was well-defined (Fig 2C). Because oyster biomass within the farm footprint was higher in summer than in fall, detailed analyses were conducted using summer data from Windmill Point. To confirm that these data were collected under flow conditions that were representative of the site, we compared current speeds from our summer sampling period to data data collected by a stationary acoustic Doppler profiler moored just outside the farm footprint from August 8 to September 8, 2017. The current speeds measured outside the farm in summer 2017 encompass a similar range and have a similar distribution to the 31-day current speed record for the site (Fig 7).

**Fig 8:**
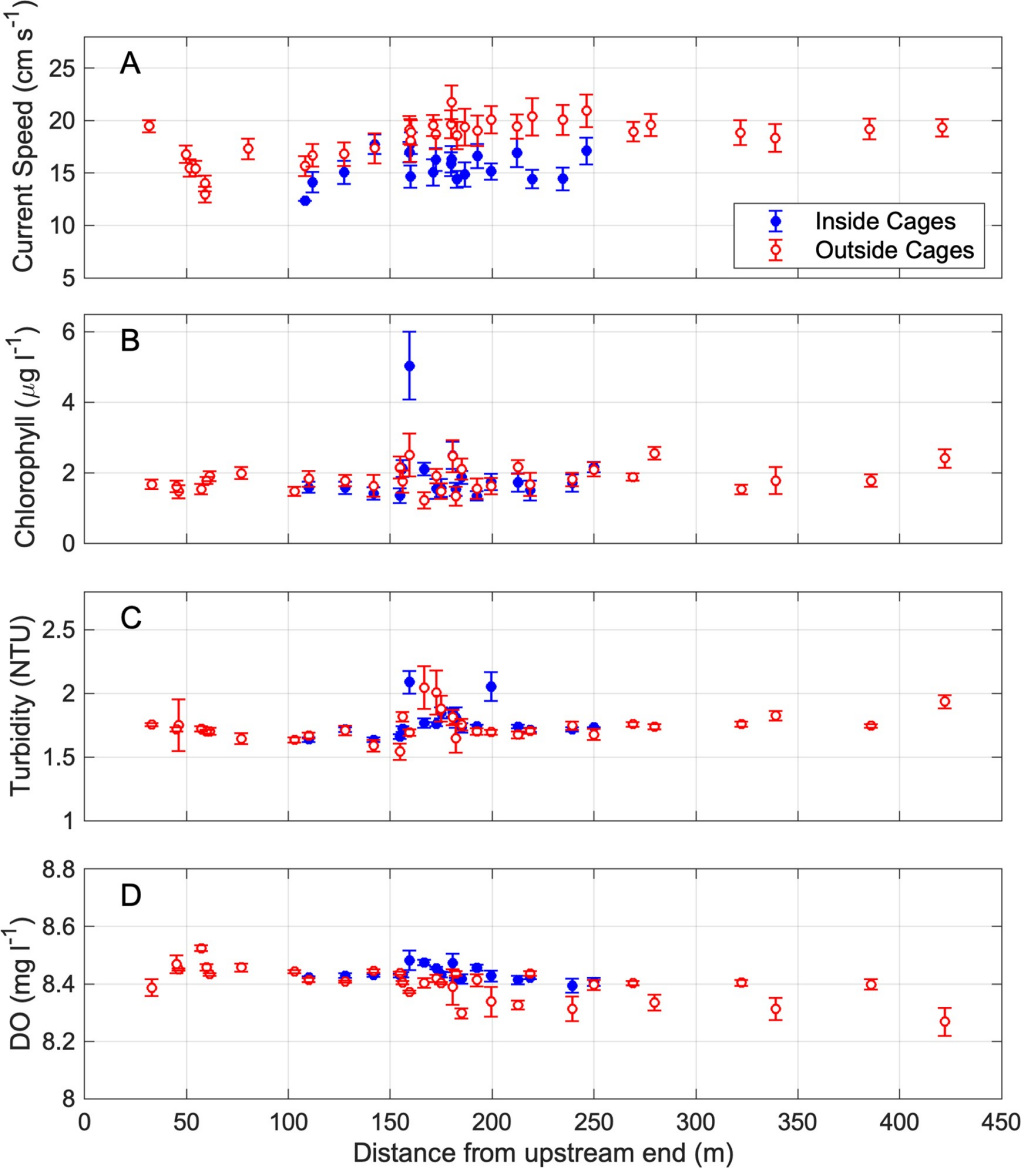
Current speed and water quality inside and outside cages by transect along distance from upstream end of sampling at Windmill Point. Data were collected at Windmill Point in summer 2017, including (A) current speed, (B) chlorophyll, (C) turbidity, and (D) DO. Open red circles indicate means of points outside the extent of cages on each transect, and filled blue circles indicate means of points inside the extent of cages on those transects that included both inside and outside points. Error bars represent ± one standard error.

With distance from the upstream end of sampling at the Windmill Point site in summer, detrended chlorophyll, turbidity, and DO did not exhibit strong changes with distance, while current speed patterns revealed a slowing of currents within the farm. On a transect-by-transect basis, inside- outside differences were minor in the context of total spatial variability (Fig 8). Current speed showed the strongest influence of the farm when examined with distance, as transect means inside cages were generally lower than transect means for points measured outside cages (Fig 8A). Effects of the farm were not as evident from water quality measurements. For one transect with measurements inside the site, chlorophyll anomalously high. However, for all other transect means, chlorophyll was nearly homogenous throughout the site. Turbidity was similarly homogenous throughout the site, with slightly higher variability in conditions on transects that passed through the farm for both inside and outside measurements (Fig 8B). DO also showed very little spatial pattern with distance through the site, showing very slightly higher DO inside cages on transects that passed through the farm (Fig 8D). Overall, spatial patterns in current speed with distance from the upstream end of sampling show a slowing of currents inside the farm, while spatial patterns in water quality show negligible influence of the farm, consistent with the results of statistical analyses.

### Overall farm effects

Overall, water quality inside and outside of farm footprints was measured during a total of six sampling periods across the different site, season, and flow speed combinations. With the exception of the farm with the smallest footprint (Bland Point) which had no significant effect on any water quality variables, oyster farms had statistically significant effects on all measured environmental variables. However, the scale of these effects was small, and the direction of effects was not consistent across farms.

Because oysters filter phytoplankton and other particles from the water column, one might expect both chlorophyll and turbidity to be lower inside the farm footprint at all sites when compared to areas outside the farm. However, across all sites and seasons, chlorophyll was never significantly lower inside than outside the farm. In contrast, turbidity was significantly lower inside the farm at Monday Creek in summer and at Broad Bay in summer and fall. Through respiration and remineralization of biodeposits, the presence of oysters has the potential to lead to decreases in DO inside farms. However, in the present study, the only significant effect on DO was an increase inside the farm at Broad Bay in summer and fall. The influence of farms on current speed was also mixed, with significantly lower speeds inside the farm at Windmill Point in both summer and fall and significantly higher speeds within the farms at Monday Creek and Broad Bay in summer.

### Conceptual farm-scale filtration calculation results

Farm-scale filtration rate calculations indicate that only a small portion of water passing through the sites around peak tidal current could be filtered by oysters. In short, a maximum of ~6% of water passing through the sites in this study within the extent of cages with a current speed of ~10 to 20 cm s^-1^ could be filtered by oysters during summer at their mean maximum filtration rate (Table 4). This is consistent with our observations of minimal impact of aquaculture farms on water quality.

**Table 4:** Conceptual filtration volume calculations for each farm.

Farm	Length (m)	Width (m)	Flow Distance (m)	Depth (m)	Current Speed (cm s^-1^)	Number of oysters	Mean ind. DW (g)	Percent of water filtered (%)
WP	150	120	150	1.5	16.8	500000	1.7	5.5
BP	80	35	50	1.5	8.8	2000	2.6	0.1
MC	80	60	60	1	8.8	117000	1	3.3
BB	320	90	130	1	11.9	136000	2.7	2.7

Percent of water filtered represents the fraction of the water passing through the extent of cages could be filtered by oysters assuming a maximum number of oysters and a maximum summer filtration rate.

## Discussion

Overall, results show statistically significant impacts of farms on local water quality but these differences are small compared to naturally occurring differences in water quality parameters attributable to differences between sites and seasons. As discussed below, some evidence suggests that aquaculture gear at some farms may damp currents and provide substrate for microalgal growth on the cage structures. Finally, simplified filtration calculations support *in situ* results, showing that oysters in these settings are likely to process only a small fraction of the water volume passing through each farm during a tidal cycle.

### Minimal water quality effects

The setting of individual farms influenced water quality far more than the presence of oysters, which showed little impact consistent with either filtration or organic enrichment. Regarding the original hypothesis, although results did show statistically significant differences between water quality measurements inside and outside of farms, those differences were too small in magnitude and too inconsistent in sign to demonstrate evidence of farm impact. Therefore, results ultimately suggest minimal water quality modification by farmed oysters at the sites in this study. The negligible impact of oysters at these sites is almost certainly due in part to the use of relatively low-density culture methods at sites with relatively high flushing rates. All farms in this study were situated in well-flushed areas with relatively short water residence times due to tidal currents and wave action. Farms in this study were also relatively low-density operations, with well-spaced cages resulting in < 60 oysters m^-2^ (Table 4). This combination of growing conditions at the sites in this study are likely beneficial for both minimizing any potentially detrimental impacts of oyster aquaculture and maximizing oyster growth.

Results showing minimal effects of these specific oyster farms on local conditions as measured here are consistent with other studies of low-density shellfish aquaculture operations in settings with sufficient hydrodynamic flow. With respect to the seabed environment, Mallet et al. [59] found that, in well-spaced operations with moderate to strong currents, effects of biodeposition on the benthos were minimal in terms of sediment redox and sulfide. In Canada, studies found little top-down control by shellfish on primary production [38,60] and minor effects overall [61]. Analogous to the results of the present study, Thorn [62] reported that separate farms were more different from one another than locations within each farm. Of the studies reviewed by Burkholder and Shumway [23], 93% found that shellfish aquaculture had a minor or negligible role in enhancing eutrophication. In short, our results support a key finding of other shellfish aquaculture studies: low-density shellfish farming at sites with relatively high flushing rates has minimal negative impact on local ecosystems [36,61,63–65].

### Hydrodynamic effects

Current damping by aquaculture gear was observed at one of the largest farms during both flood and ebb tidal periods, presenting an avenue for future research. Windmill Point was characterized by a 13 to 15 percent reduction in water column current speed within the farm footprint compared to outside (Figs 5A and 8A). Overall these results fall at the lower range of the current damping caused by a wider variety of aquaculture types in other studies (kelp-scallop, mussel, etc.), which found currents were 30 to 70 percent slower inside farms compared with outside speeds [66]. The generally lower magnitude and less consistent current damping seen in this study may be due to the differences in the scale of the gear used for various aquaculture types. The gear used in other aquaculture settings likely takes up a greater proportion of the water column than the floating and bottom cages used by oyster growers in the present study. While visiting the oyster aquaculture sites in the present study, researchers observed qualitatively that floating-cage gear also damped small wind waves (<0.25 m), though not larger wind-generated swells (> 0.25 m). Wind speed, wind-generated waves, or the reduction of wind-generated waves were not quantitatively measured as part of the present study. Reduction of wave and current energy by aquaculture gear is an important area for additional study.

### Other in-farm processes

Other water quality-related processes may have been occurring at the farms in this study, including growth of cage-associated algae. Microalgae growing on the cages may have impacted the fine-scale processes at farms, albeit without a detectable effect on water quality or sediment organic content. Increased DO was found inside one of the farms during both summer and fall, which could be due to marginally enhanced primary production. Chlorophyll was significantly higher inside the farm at the two sites sampled in fall. These differences in DO and chlorophyll, though small in magnitude, may be attributable in part to the microalgae growing on the cages and associated gear. Video footage of the underside of a cage showed that algae, likely benthic diatoms, growing on cage surface were sheared off as waves moved the bags within the cages, and the algae remained suspended in the water column. Our results do not provide a means of quantifying the scale of the contribution of cage-associated algae to our chlorophyll measurements. The relative contributions of gear-associated algae and water column phytoplankton to total primary production at aquaculture sites is an important area for future study.

The larger scale spatial differences observed in site conditions (Fig 5) may be in part due to differences in wave energy at the various sites as indicated by differences in the grain size of sediments. The relatively enclosed sites at Monday Creek and Broad Bay had the finest grain sizes and highest percentages of organic matter in bottom sediments (Table 3). Lower wave action and somewhat longer residence times likely allowed fine-grained material to accumulate over time, creating conditions that favor regular resuspension of bottom sediments at peak tidal flows. This interpretation is consistent with higher observed water column turbidity at Monday Creek and Broad Bay (Fig 5C). In contrast, Windmill Point had the coarsest bottom sediment grain size, the lowest percentage of organic matter, and low water column turbidity (Table 3; Fig 5C). The wave-exposed sites at Bland Point and Windmill Point had higher energy overall, which likely favored the removal of biodeposits and organic matter from those sites. The present study does not address impacts on the benthos, which is an important issue for future study.

While our conceptual filtration calculation estimated that the oyster farms in this study had minimal potential to modify water quality at relatively high current speeds, slower theoretical current speeds only slightly increased filtration potential. Slower current speeds, such as those experienced surrounding slack tide, may slightly increase the potential effects of oysters on water quality, but only to a certain point. In a general sense, a 50% reduction in current speed described in Table 4 effectively doubled the volume of water that could potentially be filtered by oysters (≤ ~12%). Specifically, at Windmill Point in summer 2017, current speed inside the farm for the entire time of sampling, including nearly six hours, ranged from 10.2 to 20.2 cm s^-1^, in terms of the 25^th^ to 75^th^ percentiles of measured currents speeds (Fig 7). Using these upper and lower bounds for current speed to calculate a potential filtration range, oysters at the Windmill Point farm could theoretically filter 4.6 to 9.1% of the water passing through the farm. Increased potential for water quality modification by oysters at slack tide highlights a limitation of the present study and an avenue for future research. This study may have been limited in the overall temporal scale of sampling. Each set of transects only captured one snapshot in time. The contribution of tidal stage, including slack tide, to water quality effects of oyster farms is a logical avenue for future study. Nevertheless, it is important to note that even the highest possible estimation of filtration at Windmill Point, using the lower 25^th^ percentile of all current speeds measured, yielded a low volume of water that could potentially be filtered by oysters (~9%).

In contrast, other shellfish farms showing significant water clarity modification are cultured at a greater vertical depth scale, larger spatial scale, and higher density than the oyster farms in this study. For example, mussel longline cultures use a large portion of the water column with depth and often extend over large spatial footprints, while the oyster farms in the present study took up a very small fraction of the water column and a small spatial scale in comparison. Nielsen et al. [67] found that a Danish mussel farm depleted chlorophyll by 27 to 44%. The example Danish mussel farm measured ~188,000 m^2^ in area, and mussels were cultured at a density of ~1000 individuals m^-2^, one to two orders of magnitude larger and more densely cultured than the four oyster farms in this study (2,800 to 20,000 m^2^ and cultured at a density of < ~60 individuals m^-2^). Using the metrics from Nielsen et al. [67] and a mussel filtration rate from Clausen and Riisgard [68], a conceptual filtration rate, as detailed above, was calculated for the example mussel farm. The calculation reveals that ~ 9 to 28% of the water passing through the example Danish mussel farm was able to be filtered by the organisms (with possible flow distances of 250 to 750 m, respectively). Compared to the oyster farms in the present study, this example longline mussel farm showed a higher potential modification of water clarity, consistent with the results presented by Nielsen et al. [67]. Though simplified, this comparison supports observed results and provides a global context for the relatively small impacts of the Virginia oyster farms measured in this study.

## Conclusions

This study investigated four commercial oyster farms in lower Chesapeake Bay and found minimal impacts of farms at most sites. For the water quality variables considered (chlorophyll, turbidity, and DO), effects associated with environmental setting-related differences among sites and seasons were generally an order of magnitude greater than the effects of the farms. Although large sample sizes were often able to resolve statistically significant differences in water quality inside vs. outside farms, the effects of oyster aquaculture on observed water quality variables rarely aligned with the expectation that aquaculture farms would decrease chlorophyll, turbidity and dissolved oxygen levels. The magnitudes of inside-outside water quality differences were small at all sites and seasons regardless of gear type. Results suggest that among-site differences in water quality were more closely related to differences in environmental setting, in terms of bed composition and wave exposure, than to differences in farm characteristics. A simplified calculation revealed that at the low culture densities investigated in this study, oysters in farms are only able to filter a small fraction of the water passing through each farm on a given tide.
